# The novel histone de acetylase 6 inhibitor, MPT0G211, ameliorates tau phosphorylation and cognitive deficits in an Alzheimer’s disease model

**DOI:** 10.1038/s41419-018-0688-5

**Published:** 2018-05-29

**Authors:** Sheng-Jun Fan, Fang-I Huang, Jing-Ping Liou, Chia-Ron Yang

**Affiliations:** 1 School of Pharmacy, College of Medicine, National Taiwan University, Taipei, Taiwan; 2 School of Pharmacy, College of Pharmacy, Taipei Medical University, Taipei, Taiwan

## Abstract

Alzheimer’s disease (AD) is a dreadful neurodegenerative disease that leads to severe impairment of cognitive function, leading to a drastic decline in the quality of life. The primary pathological features of AD include senile plaques (SPs) and intracellular neurofibrillary tangles (NFTs), comprising aggregated amyloid β (Aβ) and hyperphosphorylated tau protein, respectively, in the hippocampus of AD patients. Histone deacetylase 6 (HDAC6) is a key enzyme in this neurodegenerative disease, in particular, as it relates to tau hyperphosphorylation. This study aimed to investigate the protective effects and mechanism of the novel HDAC6 inhibitor, MPT0G211, using an AD model. Our results indicated that MPT0G211 significantly reduced tau phosphorylation and aggregation, the processes highly correlated with the formation of NFTs. This HDAC6 inhibitory activity resulted in an increase in acetylated Hsp90, which decreased Hsp90 and HDAC6 binding, causing ubiquitination of phosphorylated tau proteins. In addition, a significant increase of phospho-glycogen synthase kinase-3β (phospho-GSK3β) on Ser9 (the inactive form) through Akt phosphorylation was associated with the inhibition of phospho-tau Ser396 in response to MPT0G211 treatment. In AD in vivo models, MPT0G211 appeared to ameliorate learning and memory impairment in animals. Furthermore, MPT0G211 treatment reduced the amount of phosphorylated tau in the hippocampal CA1 region. In summary, MPT0G211 treatment appears to be a promising strategy for improving the AD phenotypes, including tau hyperphosphorylation and aggregation, neurodegeneration, and learning and memory impairment, making it a valuable agent for further investigation.

## Introduction

Alzheimer’s disease (AD) is one of the most common neurodegenerative diseases, accounting for more than 80% of dementia cases worldwide. AD leads to the progressive loss of mental capacity and behavior, with a functional decline in the ability to learn. Several hypotheses have been extended to explain AD, including the cholinergic hypothesis, which was the first theory. The cholinergic hypothesis posits that the primary problem is a deficit in acetylcholine, caused by the death of cholinergic neurons^1^. Another primary hypothesis is that hyperactivation of the *N*-methyl-d-aspartate (NMDA) receptor by glutamate leads to the production of free radicals and the activation of enzymes that contribute to the death of neuronal cells^2^. Accordingly, four drugs approved by the Food and Drug Administration are used to treat cognitive manifestations of AD, namely, the acetylcholine esterase inhibitors revastigmine, galantamine, and donepezil, as well as the NMDA receptor antagonist, memantine. These drugs have been revealed to reduce the progression of cognitive symptoms; however, the benefits are limited in more than half of the patients who take these drugs^2,3^. Thus new drug development for AD treatment is an urgent issue. Recent studies have focused on the amyloid and tau proteins as therapeutic targets. Although the detailed mechanisms remain unclear, present evidence suggests that the expression of these two markers may be linked^2,4^. For example, amyloid β (Aβ) not only spontaneously aggregates into the soluble oligomers, forming plaques resulting in network dysfunction, but also increases the accumulation of tau aggregates and the development of neurofibrillary tangles (NFTs), which leads to synaptic dysfunction and eventually neuronal loss^2,4^. Therefore, the recent treatment strategies have attempted to manage AD by modulating the functions of these two major proteins.

Histone deacetylase 6 (HDAC6) belongs to the HDAC family, and its structure contains a cytoplasmic anchoring domain, which mediates its stable anchorage in the cytoplasm. The enzymatic activity of HDAC6 is exerted on tubulin, heat-shock protein 90 (Hsp90), and cortactin substrates; thus it has been identified as a key regulator of the cytoskeleton and cell migration^5^. Present studies have demonstrated that HDAC6 levels significantly increase in the hippocampus and cortex of the AD brain, and tubulin acetylation is reduced in the neurons containing NFTs^6^. While the detailed mechanisms of these processes remain unknown, HDAC6 appears to be involved in the process of tau hyperphosphorylation^6,7^. Tau is predominantly expressed in neurons where its primary function is to promote microtubule stability^1,4^; however, the hyperphosphorylation of tau has been suggested to impair its ability to bind and stabilize microtubules, thus promoting tau self-assembly and aggregation^2,4^. In addition to hyperphosphorylation, tau becomes abnormally accumulated in dystrophic neuritis around senile plaques (SPs) and in NFTs of the AD brain^2^. Recent studies have indicated that HDAC6 interacts with tau in human brain tissues, and the inhibition of HDAC6 attenuates tau phosphorylation at T231, a critical regulatory site for tau function; however, it does not disrupt the HDAC6–tau interaction^6^. Furthermore, HDAC6 inhibition also appeared to downregulate Aβ aggregation^8^ and improve cognition in an AD mouse model^7^. In addition, a recent study indicated that mice lacking HDAC6 survive well and develop normally^9^, suggesting that the pharmacological inhibition of HDAC6 may not cause severe side effects. Collectively, these results suggest that HDAC6 may be a novel target of AD.

The present study investigated the effect and mechanism of the novel HDAC6 inhibitor, MPT0G211 (N-hydroxy-4-((quinolin-8-ylamino)methyl)benzamide), on neuronal protection and cognitive function of AD models. We found that MPT0G211 significantly inhibited tau phosphorylation on Ser396, Ser404, and phosphorylated tau (p-tau) aggregation. The HDAC6 inhibitory activity of MPT0G211 resulted in an increase in acetylated Hsp90, which decreased HDAC6–Hsp90 binding, and led to the ubiquitination of phosphorylated tau. In addition, a significant increase in phospho-glycogen synthase kinase-3β (phospho-GSK3β) on Ser9 (the inactive form) through Akt phosphorylation was associated with the inhibition of phospho-tau Ser396 in response to MPT0G211 treatment. In AD in vivo models, MPT0G211 treatment reduced the time spent by animals in finding the platform and closed arm in the Morris water maze and elevated plus maze test and reduced the amount of phosphorylated tau in the hippocampal CA1 region, which is related to learning and memory^10^. Furthermore, MPT0G211 appeared to be able to cross the blood–brain barrier (BBB) after oral administration. Collectively, these results demonstrate that MPT0G211 has high potential as a treatment strategy for AD.

## Results

### HDAC6 inhibition and selectivity by MPT0G211

**Fig. 1:**
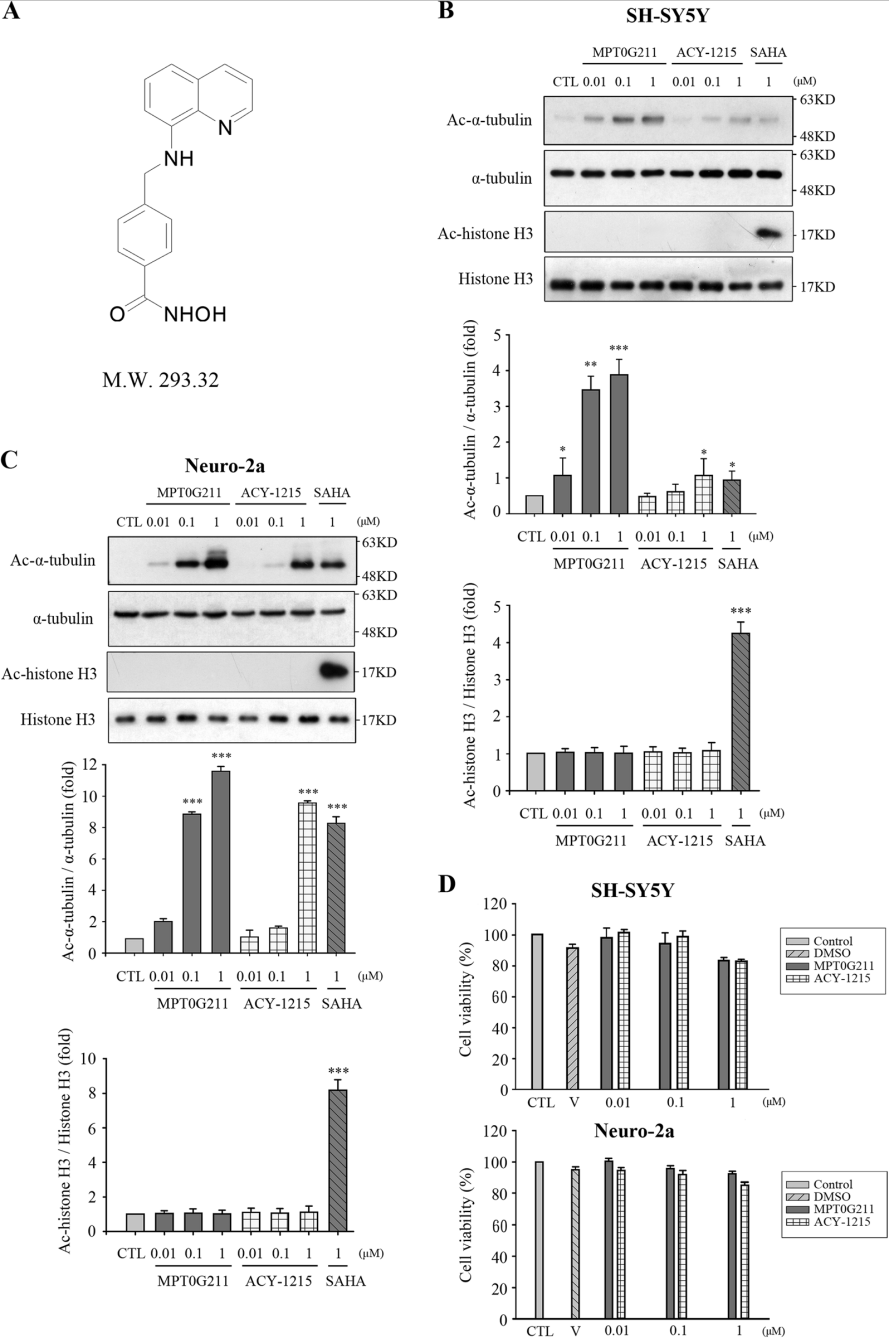
MPT0G211 exhibited potent HDAC6 inhibition. **a** The structure of MPT0G211. **b**, **c** SH-SY5Y or Neuro-2a cells were treated with the indicated concentrations of MPT0G211, ACY-1215, or SAHA for 24 h, and the whole-cell lysate was subjected to western blotting with the indicated antibodies. **d** SH-SY5Y and Neuro-2a cells were incubated for 24 h with or without the indicated concentrations of MPT0G211 or ACY-1215. Cell viabilities were determined by MTT assay. The results represent the mean ± SEM of three independent experiments; *p < 0.05, **p < 0.01 and ***p < 0.001 compared with controls

Our previous study demonstrated that MPT0G211 (Fig. 1a) exhibited potent HDAC6 inhibition (IC_50_ value = 0.291 nM) and was 1000-fold more selective for HDAC6 compared with the other HDAC isoforms^11^. In addition, MPT0G211 concentration-dependently increased the acetylation of α-tubulin in SH-SY5Y and Neuro-2a cells, the common used neuronal cell lines in the study of AD, at concentrations ranging from 0.1 to 1 μM without affecting the acetylation of histones, which is consistent with the selective inhibition of HDAC6 (Fig. 1b, c). Similar results for the acetylation of α-tubulin versus control were obtained using another HDAC6 inhibitor, ACY-1215. SAHA, a non-selective HDAC inhibitor, treatment both significantly increased the acetylation of α-tubulin and histones. Nevertheless, 1 μM ACY-1215 treatment caused less acetylation of α-tubulin; these results were consistent with MPT0G211 exhibiting more potent HDAC6 inhibition than ACY-1215^11^. In addition, MPT0G211 treatment did not result in any significant cytotoxicity in SH-SY5Y and Neuro-2a cells after 24 h (Fig. 1d), suggesting that the concentrations used in this study (0.01–1 μM) did not cause cell death.

### Inhibition of tau phosphorylation and aggregation

**Fig. 2:**
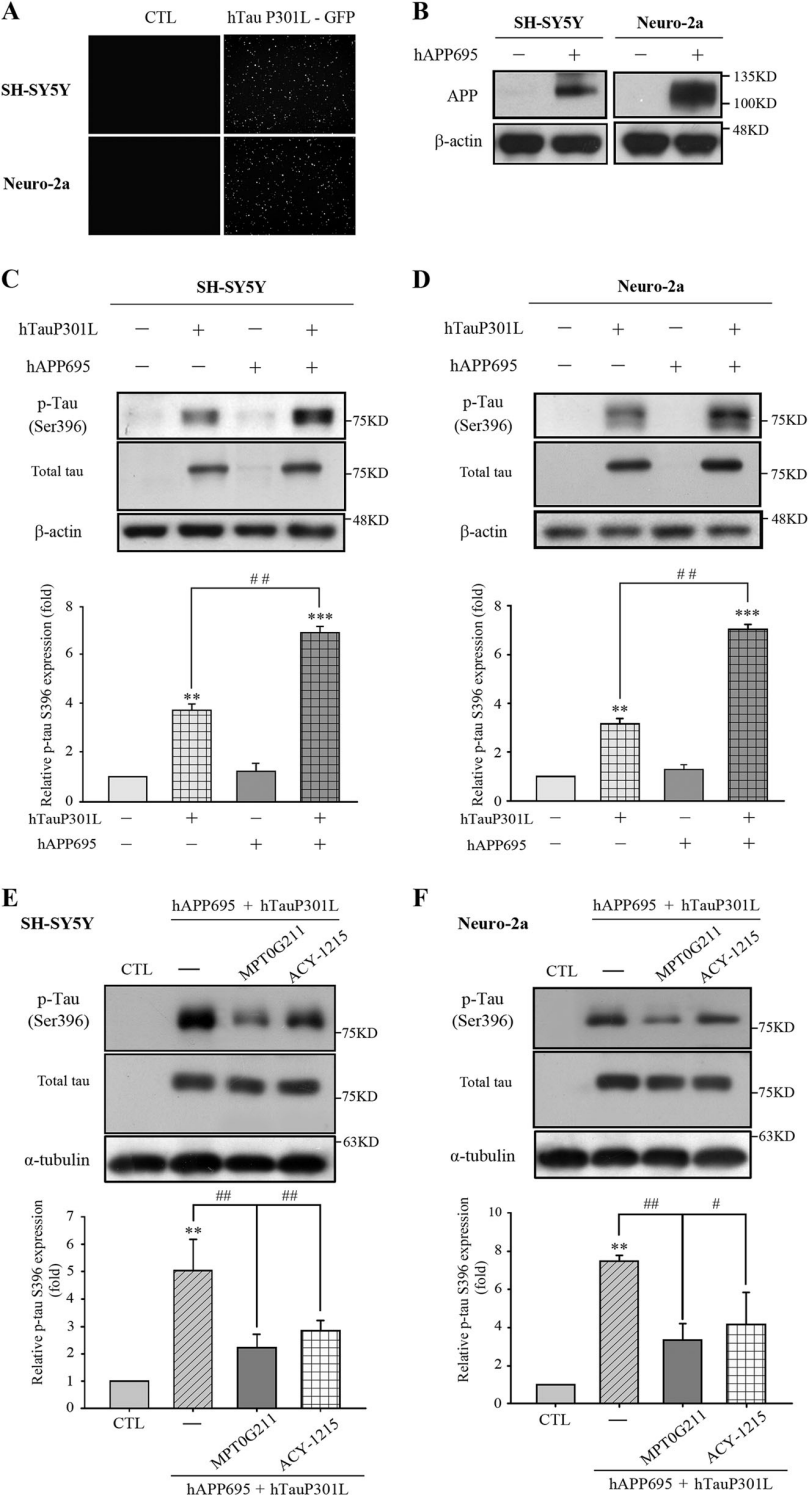
MPT0G211 significantly inhibited tau phosphorylation. **a** pRK5-EGFP-Tau P301L was used to transfect SH-SY5Y or Neuro-2a cells for 24 h; then cells were photographed by fluorescence microscopy. Images represented magnification at ×100. **b** Cells were transfected with pCAX APP 695 for 24 h, harvested, and cell lysates were subjected to Western blotting. **c**, **d** SH-SY5Y and Neuro-2a cells were transfected with pCAX APP 695 and/or pRK5-EGFP-Tau P301L for 24 h. Cell lysates were prepared for western blot analysis of the indicated proteins. **e**, **f** Cells were transfected with pCAX APP 695 and pRK5-EGFP-Tau P301L for 24 h and were then incubated with or without MPT0G211 or ACY-1215 (0.1 μM) for another 24 h. Cell lysates were subjected to western blot analysis using the indicated antibodies. Results are presented as the mean ± SEM from three independent experiments. **p < 0.01, ***p < 0.001 compared with the control group; ^#^*p* < 0.05, ^##^*p* < 0.01 compared with the indicated groups

**Fig. 3:**
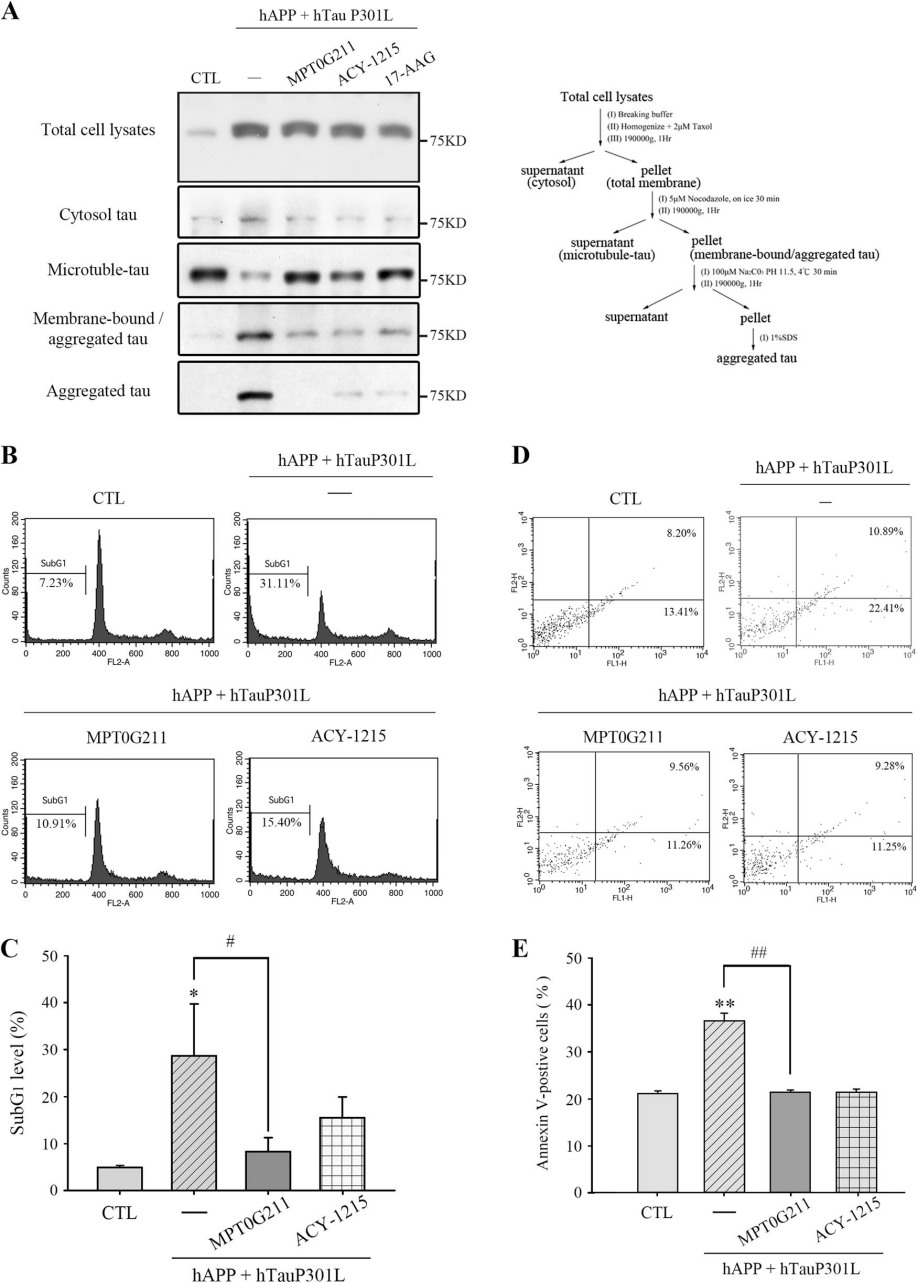
MPT0G211 significantly attenuated tau aggregation and apoptosis induced by phosphorylated tau. **a** SH-SY5Y cells were transfected with pCAX APP 695 and pRK5-EGFP-Tau P301L for 24 h and then incubated with MPT0G211 or ACY-1215 (0.1 μM) for another 24 h. The fractionation scheme used to separate different cellular pools of tau, and the western blot analysis of the effects of the compounds on tau pools generated using the fractionation scheme are presented. **b**, **d** SH-SY5Y cells were transfected for 24 h with pCAX APP 695 and pRK5-EGFP-Tau P301L, incubated with or without MPT0G211 or ACY-1215 (0.1 μM) for another 24 h, and then the cells were fixed and stained by propidium iodide (**b**) or annexin V/PI double staining (**d**) and analyzed by flow cytometry. **c**, **e** Percentages of the subG1 phase (**c**) or annexin V-positive cells (**e**) in response to drug treatment as explained in (**b**). Results are presented as the mean ± SEM. *p < 0.05 and **p < 0.01 compared with the control group; ^#^*p* < 0.05 and ^##^*p* < 0.01 compared with the indicated groups

AD is characterized by the presence of SPs, which are extracellular aggregates composed of Aβ peptides, and NFTs, which are intracellular aggregates composed of hyperphosphorylated tau^2^. In addition, Aβ can increase the accumulation of tau aggregates as well as NFTs, leading to synaptic dysfunction and eventually neuronal death^2,7^. Therefore, tau phosphorylation was measured in SH-SY5Y and Neuro-2a cells transfected with the pCAX amyloid precursor protein 695 (APP 695) plasmid with tyrosine/histidine mutations of APP and the pRK5-EGFP-Tau P301L plasmid, which encodes human mutant P301L-tau, respectively^12,13^. The efficiency of the transfections was determined by fluorescence microscopy (Fig. 2a) and western blot (Fig. 2b). The phosphorylation of tau Ser396 was significantly increased in the cells transfected with P301L, and tau was hyperphosphorylated in cells cotransfected with hAPP 695 in SH-SY5Y and Neuro-2a cells (Fig. 2c, d). Furthermore, we determined whether MPT0G211 treatment could inhibit tau phosphorylation. As reported, MPT0G211 treatment significantly inhibited the phosphorylation of tau Ser396 in both cell lines (Fig. 2e, f). ACY-1215 treatment exhibited a similar inhibition; however, it was less efficient than MPT0G211. It is known that hyperphosphorylation also increases the capacity of tau assembles to form aggregates from oligomers to fibrils, eventually leading to their deposition as NFTs and causing neuronal dysfunction, including reduced mitochondrial respiration, altered mitochondrial dynamics, and impaired axonal transport^14^. Accordingly, we used an established method to determine whether MPT0G211 reduces the polymerization of phosphorylated tau^15^; 17-AAG (17-allylamino-17-demethoxygeldanamycin), an inhibitor of Hsp90, was used as positive control. As demonstrated, cytosolic levels of tau associated with membranes and, in aggregates, were significantly increased in SH-SY5Y cells cotransfected with hAPP 695 and hTau P301L (Fig. 3a). Treatment of the transfectants with MPT0G211 significantly reduced the levels of aggregated tau (Fig. 3a). In addition, using flow cytometry to detect sub-G1 peak and annexin V-positive cells in apoptosis, we observed that MPT0G211 treatment significantly inhibited the plasmid transfection-induced sub-G1 population and annexin V-positive apoptotic cells increase in the neuronal cells (Fig. 3b–d). These results suggest that MPT0G211 not only inhibited tau phosphorylation and aggregation but also downregulated tau aggregation associated with the neuronal cell apoptosis.

### Neuroprotective effects of MPT0G211

**Fig. 4:**
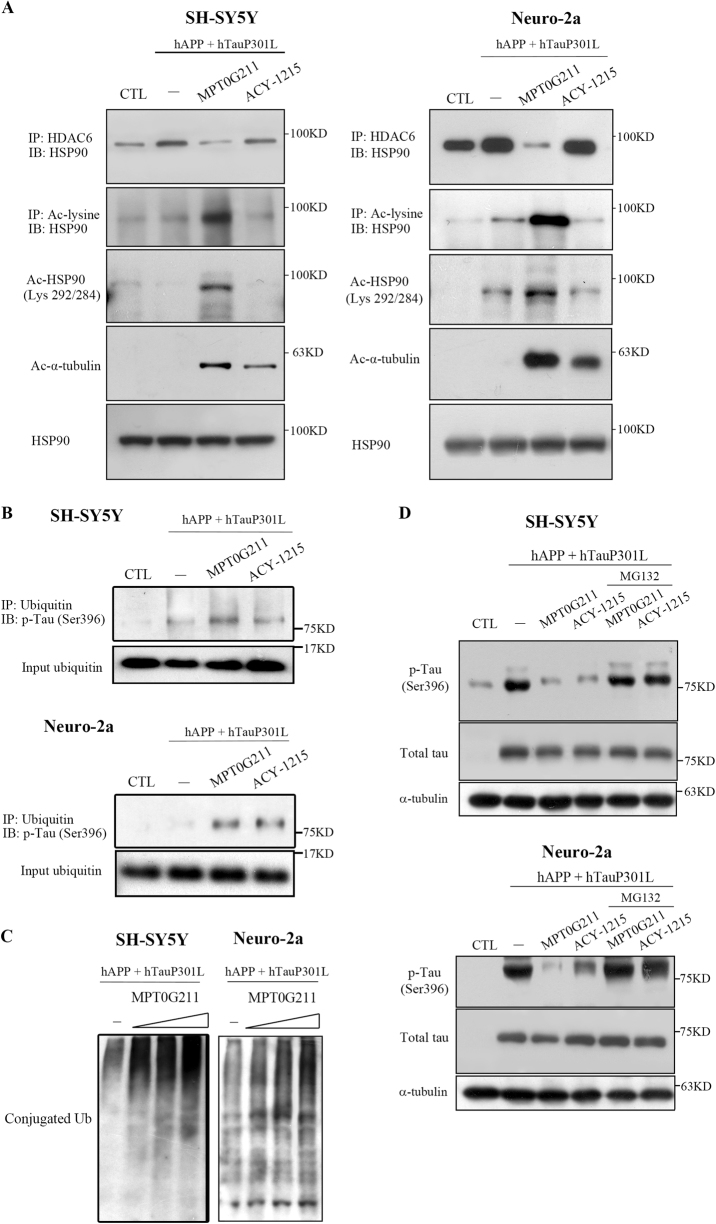
MPT0G211 increased the ubiquitination of p-tau and degradation by proteasome. **a**, **b** SH-SY5Y and Neuro-2a cells were transfected for 24 h with pCAX APP 695 and pRK5-EGFP-Tau P301L and incubated with MPT0G211 or ACY-1215 (0.1 μM) for another 24 h, after which the cell lysates were immunoprecipitated with antibodies against HDAC6, acetyl-lysine (**a**) or ubiquitin (**b**) and were subjected to immunoblotting. **c** Cells were transfected with pCAX APP 695 and pRK5-EGFP-Tau P301L for 24 h and were then incubated with MPT0G211 (0.01–1 μM) for another 24 h. Immunoblots reveal polyubiquitin complexes. **d** pCAX APP 695 or pRK5-EGFP-Tau P301L were used to transfect the cells for 24 h, incubated with or without MG132 (1 μM) for 30 min, and were then treated with MPT0G211 or ACY-1215 (0.1 μM) for another 24 h. Cell lysates were prepared for western blot analysis of the indicated proteins

As previously described, a primary pathologic component of AD is the formation of NFTs composed of hyperphosphorylated tau. Thus promoting the removal of these p-tau species may be a relevant therapeutic strategy. Although the significance of the chaperones that interplay in AD pathology remains unclear, increasing evidence suggests that Hsp–ubiquitin–proteasome system (UPS)-mediated aggregate clearance plays a pivotal role in the AD pathology and further emphasizes the role of Hsp90 as a mediator of tau protein regulation and degradation^16^. Since Hsp90 is a substrate of HDAC6, the downregulation of the expression or activity of HDAC6 has been reported to promote Hsp90 acetylation, thereby favoring tau degradation^17,18^. Therefore, we explored the neuroprotective mechanism of MPT0G211 and whether it acts through Hsp90 and UPS modulation. As indicated, plasmid cotransfection significantly increased HDAC6/Hsp90 binding (Fig. 4a). In addition, MPT0G211 treatment clearly enhanced the acetylation of Hsp90, which caused the downregulation of HDAC6/Hsp90 binding (Fig. 4a). In cells treated with MPT0G211, coimmunoprecipitation of ubiquitin with p-tau (Ser396) clearly increased (Fig. 4b), and the level of polyubiquitinated proteins significantly accumulated (Fig. 4c). In addition, proteasome inhibitor MG132 treatment significantly reversed the MPT0G211-induced inhibition of p-tau at Ser396 in both cell lines (Fig. 4d). These results suggest that MPT0G211 inhibited HDAC6/Hsp90 binding and caused subsequent proteasomal degradation of polyubiquitinated proteins.

**Fig. 5:**
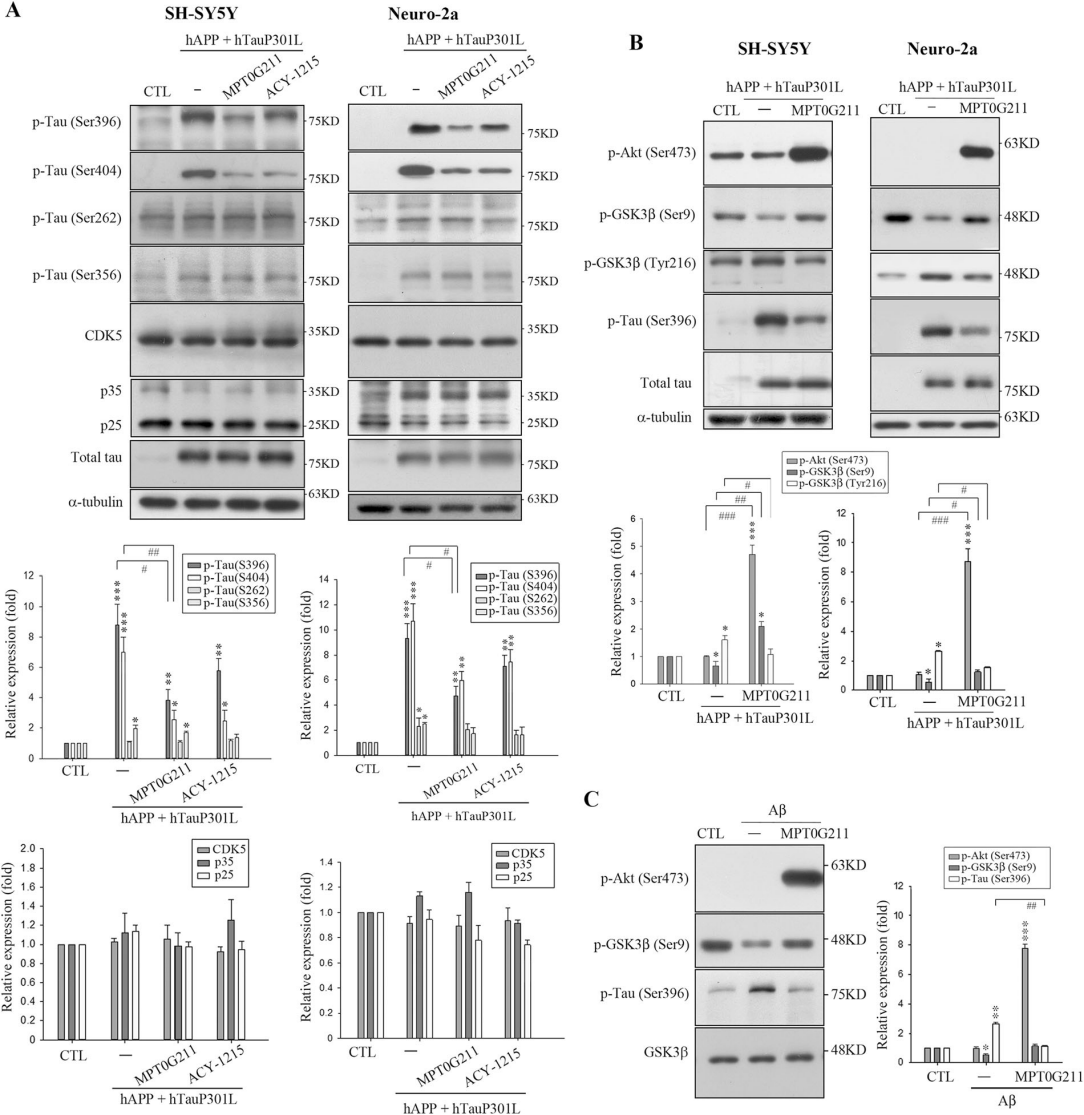
MPT0G211 significantly decreased the phosphorylation of tau by GSK3β inactivation. **a**, **b** SH-SY5Y and Neuro-2a cells were transfected for 24 h with pCAX APP 695 and pRK5-EGFP-Tau P301L and incubated with MPT0G211 or ACY-1215 (0.1 μM) for another 24 h, after which the cell lysates were subjected to immunoblotting. **c** SH-SY5Y cells were incubated with or without Aβ_1–40_ (10 μM) for 24 h and then with MPT0G211 (0.1 μM) for a further 24 h. Cell lysates were prepared for western blot analysis of the indicated proteins. Results are presented as the mean ± SEM. *p < 0.05, **p < 0.01, and ***p < 0.001 compared with the control group; ^#^*p* < 0.05, ^##^*p* < 0.01, ^###^*p* < 0.001 compared with the indicated groups

Moreover, we identified whether MPT0G211 also contributed to the attenuation of tau phosphorylation. Previous studies have indicated that GSK3β and cyclin-dependent kinase 5 (CDK5) are tau kinases, and these proteins have been proposed to contribute to the pathogenesis of AD^19,20^. In addition, the activity of GSK3β is dependent on its phosphorylation at specific sites, including Ser9 and Tyr216, which can inhibit or increase GSK3β activity, respectively^19^. Therefore, we evaluated CDK5 and phospho-GSK3β on Ser9 and Tyr216 expression in response to plasmid cotransfection and MPT0G211 treatment. As demonstrated, phosphorylation of tau Ser396 and Ser404 significantly increased in the cells transfected with the plasmids, but no increases or mild increases in the phosphorylation of other sites (Ser262 and Ser356) were observed (Fig. 5a). MPT0G211 treatment significantly attenuated the phosphorylation of tau Ser396 and Ser404 in both cell lines (Fig. 5a); no marked changes in CDK5, p25, or p35 expression were associated with MPT0G211-induced phospho-tau downregulation; however, significant phospho-GSK3β increases on Ser9 through Akt phosphorylation were associated with the inhibition of phospho-tau Ser396 in response to MPT0G211 treatment (Fig. 5b). In addition, a decrease in phospho-GSK3β on Ser216 was also observed. Accumulating evidence suggests that Aβ promotes tau phosphorylation through several mechanisms, including the activation of GSK3β^21^. Therefore, tau phosphorylation was measured in both cell lines treated with Aβ_1–40._ As reported, Aβ_1–40_-induced significant p-GSK3β (Ser9) attenuation and increase in p-tau (Ser396); MPT0G211 treatment diminished GSK3β activity and Aβ_1–40_-induced tau phosphorylation (Fig. 5c).

### MPT0G211 ameliorates learning and memory impairments in an animal model

**Fig. 6:**
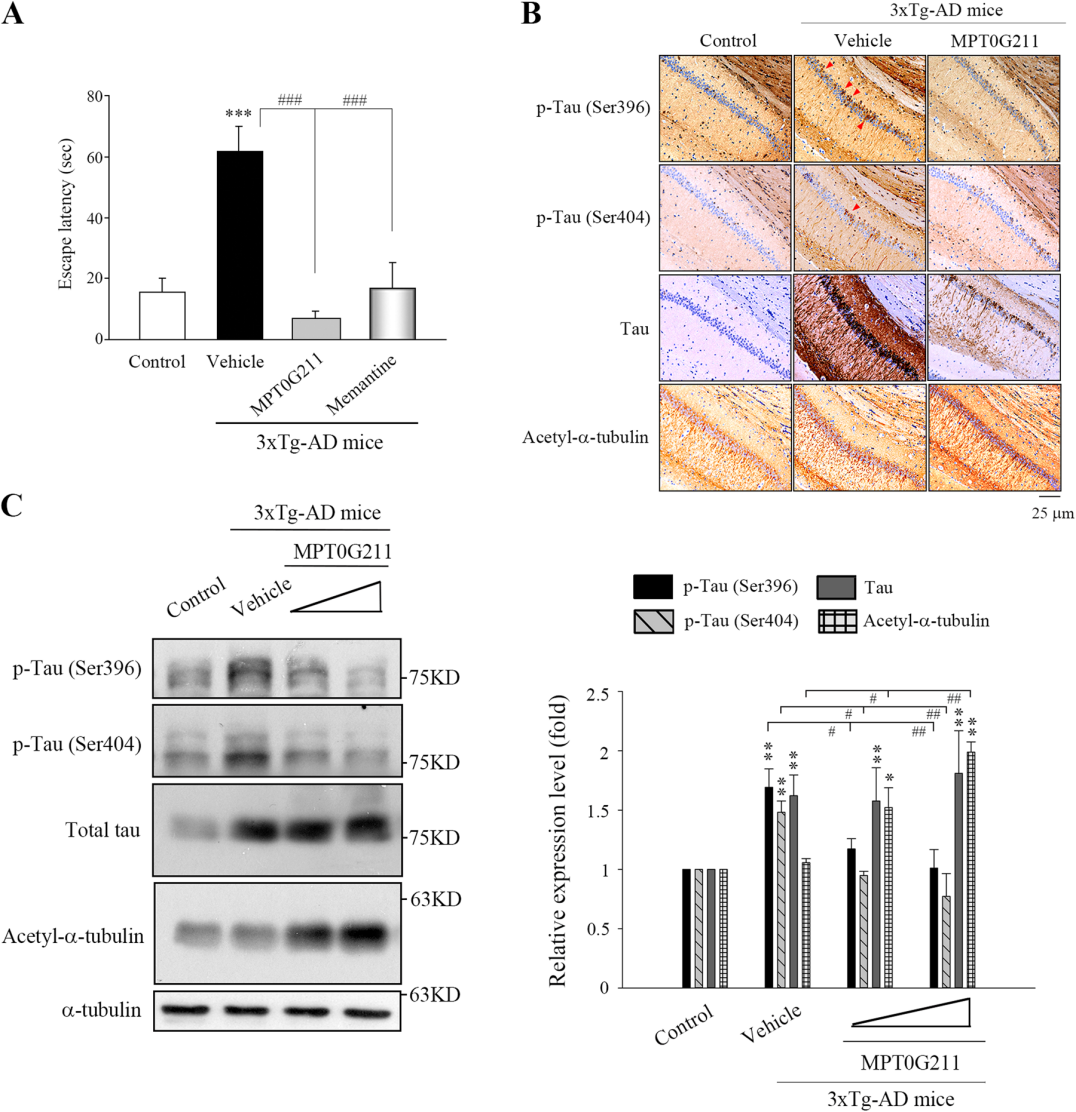
MPT0G211 significantly ameliorated the spatial memory impairment in vivo. **a** 3×Tg-AD mice (aged 6 months) were orally administered MPT0G211 (50 mg/kg) or memantine (30 mg/kg) daily for 3 months; mice were then subjected to the Morris water maze, and their escape latency times were measured. **b**, **c** 3×Tg-AD mice were orally administered MPT0G211 (50, 100 mg/kg) for 3 months, then mice were sacrificed and their brains were removed for immunohistochemical analysis for acetyl-α-tubulin expression and tau phosphorylation (Ser396, Ser404) in the CA1 region of the hippocampus (**b**) and western blot analysis (**c**). Red arrowheads indicate phosphorylated-tau proteins. Scale bar = 25 μm. Data represent the mean ± SEM. *p < 0.05, **p < 0.01, and ***p < 0.001 compared with the basal or control group; ^#^*p* < 0.05, ^##^*p* < 0.01, and ^###^*p* < 0.001 compared with the vehicle-treated group

We further evaluated whether MPT0G211 treatment ameliorates learning and memory impairment. Using the Morris water maze test, we first evaluated the learning ability by training rats with a hidden platform. Triple transgenic (3×Tg-AD) mice, which harbor APP_Swe_ and tau_P301L_ mutant transgenes, are known to develop neuropathologies, such as plaque and tangles^19^. In this study, we used these mice to evaluate the neuroprotective effect of MPT0G211, and memantine (a drug that is approved for the treatment of moderate-to-severe AD) was used as reference compound. Oral administration of MPT0G211 significantly ameliorated memory impairment (Fig. 6a). Immunohistochemical analysis revealed that mutant transgenes mediated increases in total tau and tau phosphorylation at Ser396/Ser404 in the hippocampal CA1 region of mice that harbor tau_P301L_ mutant transgene; MPT0G211 treatment significantly downregulated p-Tau (S396) and p-Tau (S404) expression and increased acetyl-α-tubulin accumulation in the 3×Tg-AD mice brain (Fig. 6b). Western blot analysis also demonstrated similar results (Fig. 6c). These results provide strong support that MPT0G211 treatments can ameliorate Alzheimer’s deficits.

**Fig. 7:**
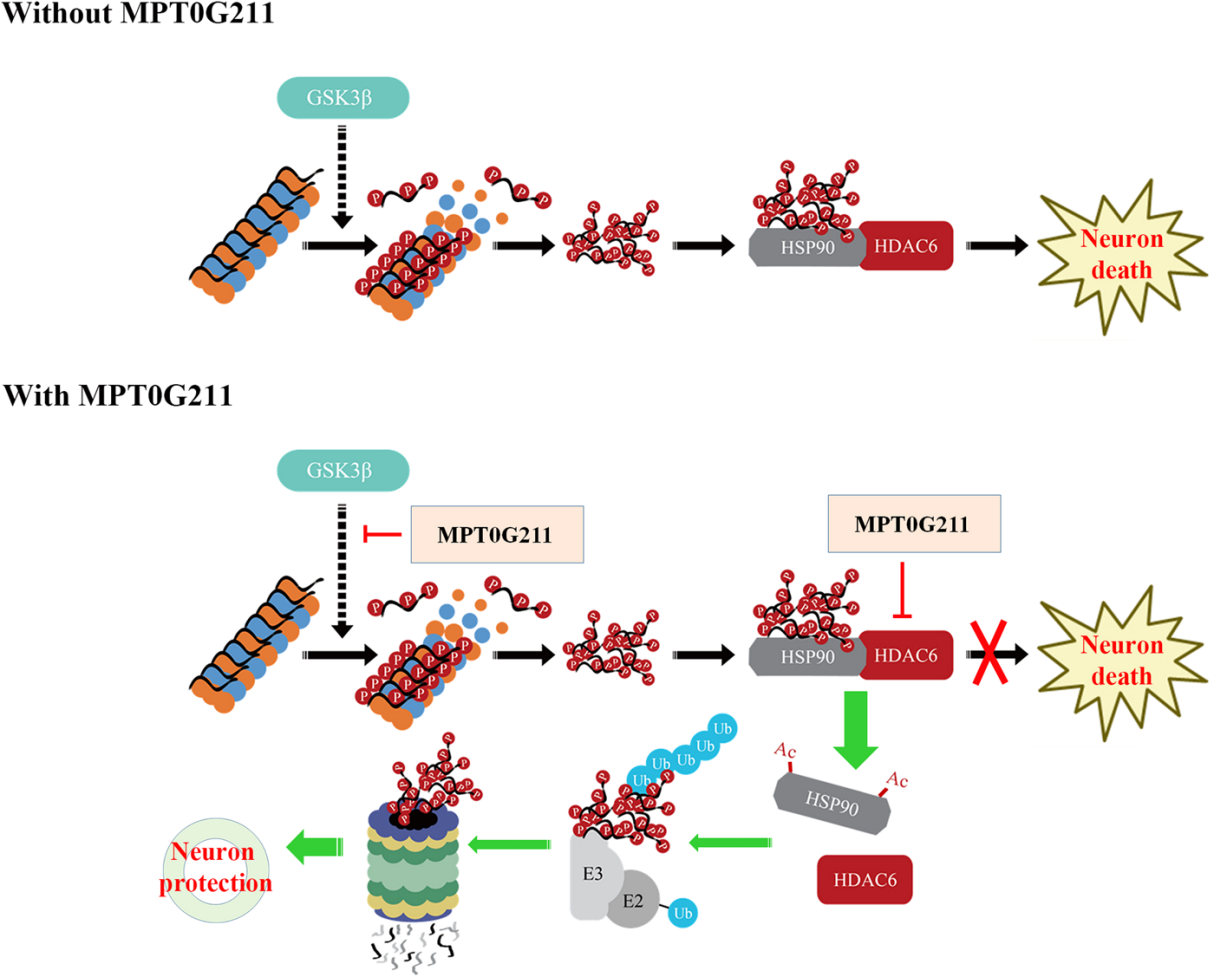
Summary of the proposed mechanism of the neuroprotective effect of MPT0G211. Tau phosphorylation can be facilitated by GSK3β, lead to p-tau aggregation, and cause neuron death. MPT0G211 treatment not only diminished tau phosphorylation by inhibition GSK3β activity but also enhanced the acetylation of Hsp90, which caused the downregulation of HDAC6/Hsp90 binding and facilitated proteasomal degradation of polyubiquitinated p-tau

Successful crossing of the BBB by therapeutic drugs is essential in the treatment of CNS disorders. To explore whether MPT0G211 can penetrate into the brain, single oral administration of MPT0G211 was performed, and plasma and brain samples were collected and analyzed by liquid chromatography tandem mass spectrometry (LC-MS/MS) to obtain brain/plasma ratios. As observed, MPT0G211 was detected in brain samples 1 h after administration, and the brain/plasma ratio was 1.01 (Table 1). Concentrations of MPT0G211 were detected in the brain for at least 3 h. These results clearly indicated that MPT0G211 can penetrate the BBB, where it potentially ameliorates learning and memory deficits. A summary of the proposed neuroprotective mechanism of MPT0G211 is illustrated in Fig. 7.

**Table 1:** Brain and plasma concentrations of MPT0G211 after oral administration in rats

Compound	Route	Dose (mg/kg)	Time (h)	Brain concentration (ng/g)	Plasma concentration (ng/g)	Brain/plasma ratio
MPT0G211	PO	50	1	56.9 ± 3.4	56.4 ± 4.0	1.01
	PO	50	3	11.0 ± 0.1	12.2 ± 1.4	0.90

## Discussion

Hyperphosphorylated tau can aggregate into filaments, and these tau filaments can continue to aggregate and form insoluble deposits, which are referred to as NFTs. This accumulation of abnormal proteins results in neurotoxicity in the brain, leading to AD. Thus inhibiting tau phosphorylation and promoting phosphorylated tau degradation remain the main goals of AD treatment^2^. Previous studies have demonstrated that HDAC6 is involved in the process of tau hyperphosphorylation^6,7^, and increased HDAC6 levels are found in the hippocampus and cortex of the AD brain^6^. Reducing HDAC6 activity has been reported to ameliorate cognitive deficits in an AD mouse model^7,17^. Mice lacking HDAC6 survive well and develop normally^9^, suggesting that pharmacological inhibition of this enzyme may not cause severe side effects. Collectively, these results suggest that HDAC6 may be a novel, promising target of AD.

APP 695 is a plasma membrane protein known to be the source of Aβ^12,22^. Recent studies have indicated that significant Aβ_1–40_ and Aβ_1–42_ can be detected in APP 695-overexpressing neuronal cells, and Aβ can increase the tau hyperphosphorylation and decrease the solubility of tau^23,24^. In addition, transfecting tau P301L into cells results in proteins that are more favorable substrates for phosphorylation by protein kinases, increases tau phosphorylation, and also dramatically enhances the tendency for aggregation and polymerization into filaments, with resulting neuronal dysfunction^13,17,25^. In contrast, reducing the phosphorylation of tau at Ser199/Ser202 and Ser396/Ser404 appears to restore the tau-microtubule assembly^26^. In addition, by using different phosphorylated tau variants, it has been found that p-tau is toxic to the cultured cells^27^.An animal model for tauopathy has also revealed that p-tau is the primary factor for cellular toxicity^28^. In this study, the cells co-transfected with hAPP 695 and hTau P301L resulted in significant increases in the phosphorylation of tau Ser396/Ser404 (Fig. 5a) and aggregation (Fig. 3a), which then resulted in apoptosis (Fig. 3b–d). Our results also revealed that MPT0G211 treatment not only significantly inhibited the tau phosphorylation of tau Ser396/Ser404, aggregation, and subsequent apoptosis, but also penetrated the BBB (Table 1) and ameliorated learning and memory impairment in an animal model (Fig. 6).

The UPS is responsible for degrading the majority of cellular proteins and maintaining protein homeostasis. During this process, molecular chaperones act as specialized machinery for assisting protein folding. A recent study has indicated that molecular chaperones may also be involved in AD pathology, and increasing evidence indicates that Hsp–UPS-mediated aggregate clearance is the pivotal mechanism of AD treatment^16^. Hsp90 is a major Hsp that interacts with diverse proteins, including tau, and previous studies have indicated that hyperphosphorylated tau can be recognized by Hsp90^17,29^. Following binding with Hsp90, the specific components of the Hsp90 complex determine whether the client proteins enter a refolding pathway or are targeted for degradation by the UPS^17^. In the ADP-bound conformation, Hsp90 associates with the client-bound Hsp70/Hsp40 complex and recruits the ubiquitin ligase carboxy terminus of Hsp70-interacting protein, directing the client to proteasomes for degradation^16^. Replacement of ADP with ATP can alter Hsp90 conformation, resulting in the release of Hsp70/Hsp40 and allowing the recruitment of p23, which can stabilize the client proteins^16^. A recent study has indicated that the acetylation state of Hsp90 modulates the function of Hsp90^30^. For example, hyperacetylated Hsp90 reduces the affinity of Hsp90 binding to the protein complex, which leads to impairment in the chaperone function and promotes client protein degradation^17,18,30^. Bali and Nimmanapalli groups have demonstrated that depletion of HDAC6 levels, or the inhibition of its deacetylase activity, results in hyperacetylation of Hsp90 associated with inhibition of ATP and client protein binding to Hsp90, eventually leading to polyubiquitylation and proteasome degradation of client proteins^31,32^. In this study, MPT0G211 treatment significantly increased the hyperacetylation of Hsp90 and p-tau (Ser396) ubiquitination (Fig. 4a, b). In addition, proteasome inhibitor MG132 treatment clearly reversed the inhibition of p-tau caused by MPT0G211 (Fig. 4d). These results are consistent with the previous studies and indicate that MPT0G211 augments the polyubiquitination of p-tau and subsequent degradation.

We also evaluated whether MPT0G211 plays a role in the modulation of kinase activities involved in the phosphorylation of tau. Among numerous kinases that have been implicated in tau phosphorylation, GSK3β and CDK5 have been identified as prime candidates for aberrant tau hyperphosphorylation at disease-associated sites^19,20^. In this study, inhibition of p-tau Ser396 by MPT0G211 treatment was not associated with changes in the CDK5 expression (Fig. 5a); however, it was associated with phospho-GSK3β increasing on Ser9 (Fig. 5b). GSK3β is a constitutively active protein kinase and its regulation is primarily based on the inhibition of its activity by phosphorylation on Ser9^19^; activation of the phosphoinositide-3 kinase (PI3K)/Akt pathway has been identified to suppress GSK3β activity via Ser9 phosphorylation by Akt^33^. GSK3β activity is increased in the brain of AD patients, and overexpression of GSK3β in mice results in tau hyperphosphorylation and AD-like tau pathology^34,35^. Phosphorylation of tau by GSK3β occurs in the regions surrounding the microtubule-binding domain, whereas phosphorylation at these sites has been found to cause tau detachment from microtubules and lead to self-aggregation^36^. In addition, a previous study has demonstrated that exposure of neurons to Aβ increases GSK3β activity through the inhibition of PI3K signals, and blockade of GSK3β activity can prevent Aβ-induced neurodegeneration^37^. Herein, significant p-GSK3β increases on Ser9 through Akt phosphorylation were associated with p-tau Ser396 in response to MPT0G211; another p-GSK3β on Ser216 decreasing was also observed (Fig. 5b). In addition, our results also demonstrated that MPT0G211 treatment diminishes GSK3β activity in Aβ_1–40_-induced tau phosphorylation (Fig. 5c). Furthermore, we also revealed that MPT0G211 can penetrate the BBB (Table 1), and oral administration of MPT0G211 significantly ameliorated memory impairment and tau phosphorylation (Fig. 6). Collectively, our novel results suggest that MPT0G211 has a high potential as an AD treatment agent.

## Materials and methods

### Cell lines

The human neuroblastoma cell line SH-SY5Y, kindly provided by Professor Shiow-Lin Pan (Ph.D. Program for Cancer Molecular Biology and drug Discovery, Taipei Medical University), was maintained in Ham’s F12 nutrient mixture/Minimum essential media with 10% fetal bovine serum, penicillin (100 units/mL), and streptomycin (100 μg/mL). The mouse neuroblastoma cell line Neuro-2a, which was purchased from the Bioresource Collection and Research Center (Hsinchu, Taiwan), was cultured in Minimum essential media containing 10% fetal bovine serum, penicillin, and streptomycin. All cell lines were incubated in an atmosphere containing 5% CO_2_ at 37 °C.

### Materials

MPT0G009 and SAHA were synthesized by Professor Jing-Ping Liou to >98% purity^11^. Primary antibodies against APP, acetyl-histone 3, histone 3, α-tubulin, acetyl-α-tubulin, Hsp90, HDAC6, acetyl-lysine, p-Akt (Ser473), p-GSK3β (Ser9), and p-GSK3β (Tye216) were purchased from Cell Signaling Technology (Danvers, MA, USA). Antibodies to p-tau (Ser396) and p-tau (Ser404) were purchased from Abcam (Cambridge, MA, USA); antibodies to p-tau (Ser262) and p-tau (Ser356) were obtained from Thermo Fisher Scientific (Waltham, MA, USA). An antibody against ubiquitin was purchased from Santa Cruz Biotechnology, Inc. (Dallas, TX, USA). Aβ_1–40_ was purchased from AnaSpec (Fremont, CA, USA). The labeled secondary antibodies were horseradish peroxidase (HRP)-conjugated anti-mouse and anti-rabbit IgG antibodies (Jackson ImmunoResearch Inc., West Grove, PA, USA). The pCAX FLAG APP and pRK5-EGFP-Tau P301L plasmids were provided by Dennis Selkoe and Tracy Young-Pearse (Addgene plasmid #30154) and Karen Ashe (Addgene plasmid #46908), respectively. TurboFect transfection reagent was from Fermentas (Burlington, Ontario, Canada). ACY-1215 was purchased from BioVision Inc. (Milpitas, CA, USA). Unless otherwise stated, all other chemicals were purchased from Sigma-Aldrich (St. Louis, MO, USA).

### Transfection assay

Cells were seeded 1 day before transfection. The plasmids pCAX FLAG APP and pRK5-EGFP-Tau P301L (1 μg each) and 1 μL of TurboFect transfection reagent were mixed for 20 min at room temperature, added to the cells, and the suspensions were incubated for 24 h at 37 °C in a humidified atmosphere containing 5% CO_2_.

### Flow cytometry

After drug treatment, the cells were collected, washed with cold phosphate-buffered saline (PBS), and fixed with 75% alcohol overnight at −20 °C. After centrifugation, the fixed cells were washed with cold PBS and resuspended in DNA extraction buffer (0.2 M Na_2_HPO_4_, 0.1 M citric acid, pH 7.8) for 30 min. The cells were centrifuged and incubated with propidium iodide (PI) (0.1% Triton X-100, 100 μg/mL RNase A, and 80 μg/mL PI in PBS) for 30 min. The cell cycle was analyzed using a FACScan Flow cytometer and Cell Quest software (Becton Dickinson, Mountain View, CA, USA).

### Immunoblot and immunoprecipitation analyses

Cells (1 × 10^6^) were incubated for 10 min at 4 °C in lysis buffer (20 mM HEPES, pH 7.4, 2 mM EGTA, 50 mM β-glycerophosphate, 0.1% Triton X-100, 10% glycerol, 1 mM dithiothreitol, 1 μg/mL leupeptin, 5 μg/mL aprotinin, 1 mM phenylmethylsulfonyl fluoride, and 1 mM sodium orthovanadate), scraped from the plate, incubated on ice for 10 min, and centrifuged at 17,000 × *g* for 30 min at 4 °C. Protein samples (30 μg) were electrophoresed through sodium dodecyl sulfate-polyacrylamide gels (SDS-PAGE) and transferred onto a nitrocellulose membrane, which was then blocked by incubation for 30 min at room temperature with 5% fat-free milk in PBS. Immunoblotting was performed by overnight incubation at 4 °C with primary antibodies in PBS, followed by incubation for 1 h at room temperature with HRP-conjugated secondary antibodies. Bound antibodies were measured using ECL reagent (T-Pro Biotechnology, New Taipei City, Taiwan), and the membrane was placed on a photographic film. Cell lysates (30 μg) were incubated with antibodies (1 μg each) and protein A/G agarose beads overnight at 4 °C. The precipitated beads were washed three times with 1 mL of ice-cold cell lysis buffer, and bound immune complexes were separated using 8% SDS-PAGE, followed by immunoblotting using the indicated primary antibody.

### Subcellular fractionation

This assay followed a published method^6^. Briefly, cells (1 × 10^7^) were treated with drugs for 24 h and scraped off into breaking buffer (0.25 M sucrose, 10 mM HEPES, pH 7.2, 1 mM MgAc_2_, and protease inhibitors). The lysate was centrifuged at 190,000 × *g* for 1 h, and the supernatant was collected as the cytosolic fraction. The pellet was resuspended and incubated with 5 μM nocodazole on ice for 30 min and then centrifuged for 1 h at 190,000 × *g*. The supernatant contained microtubule-tau, and the pellets contained membrane-bound and aggregated tau. The pellets were further extracted using 100 mM sodium carbonate buffer, pH 11.5, centrifuged at 190,000 × *g* for 1 h, and washed with 1% SDS to produce a fraction containing tau aggregates. Samples containing equal amounts of protein were analyzed using SDS-PAGE.

### Analysis of cognitive dysfunction

Six-month-old female B6;129-*Psen1*^*tm1Mpm*^ Tg(APPSwe,tauP301L)1Lfa/Mmjax (3xTg-AD) mice and control B6129SF2/J mice were obtained from the Jackson Laboratory (Bar Harbor, ME, USA), orally administered MPT0G211 (50 mg/kg) or memantine (30 mg/kg, dissolved in water) once daily for 3 months, and then subjected to Morris water maze. The assay that using 3xTg-AD mice was entrusted to carry out by Development Center for Biotechnology (New Taipei City, Taiwan).

### Morris water maze

The water maze was constructed as a white circular pool filled with water, and the water temperature was maintained at 23–27 °C. A white platform was established and submerged 5 cm below the surface of the water and styrofoam beads were added to make the platform invisible. Animals were trained by permitting them to stay for 10 s upon reaching the platform. If the animals failed to locate the platform in 180 s, it was placed on the platform for 10 s to learn and memorize the location of the platform. Training was performed twice daily and 4 days during 1 week. Test trials were repeated twice with a 20 min interval for each animal. Scopolamine (1 mg/kg) was injected intraperitoneally 90 min before the test trial to induce AD-like features, except for the control group.

### Elevated plus maze

The plus maze consisted of two open and two closed arms, connected by a central platform. Animals were individually placed at the end of either of the open arms facing away from the central platform. The time for each rat to move from the open arm to either of the closed arms was recorded. If the rat did not enter the closed arm within 180 s, it was gently pushed into the closed arm and assigned a transfer latency equal to 180 s. These experiments were performed in accordance with relevant ethics guidelines and regulations, which were reviewed and approved by the Animal Use and Management Committee of the College of Medicine, National Taiwan University (IACUC number: 20130361).

### BBB crossing assay

This assay was performed by Eurofins Scientific. Briefly, SD rats weighing 200–300 g were provided by BioLasco Taiwan (under the license of Charles River Laboratories). Rats were sedated under general inhalant anesthesia (3% isoflurane) for blood collection using cardiac puncture 60 and 180 min after oral administration of the test compound. Aliquots of blood were gently mixed with lithium heparin, kept on ice, and centrifuged at 2500 × *g* for 15 min at 4 °C. The plasma was then harvested and stored at −70 °C. After blood sampling, rats were decapitated, and the entire brain was quickly removed and rinsed with cold saline (0.9% NaCl, w/v). The surface vasculature was ruptured, blotted with dry gauze, weighed, and stored on ice within 1 h of collection. Each brain was homogenized in 3 mL cold PBS, pH 7.4, for 10 s on ice, and centrifuged at 5400 × *g* for 15 min at 4 °C. Supernatants were precipitated using acetonitrile precipitation and subjected to high-performance LC-MS/MS.

### Data analysis and statistics

Each result represents the mean ± SEM of at least three independent experiments. The data were analyzed using the Student’s *t*-test. One-way analysis of variance was performed to analyze the animal data. Parameters with a *p*-value < 0.05 were considered statistically significant.
